# Supplementary material for: Forecasting Postoperative Delirium in Older Adult Patients with Fast-and-Frugal Decision Trees
Source: J Clin Med. 2022 Sep 24;11(19):5629. doi: 10.3390/jcm11195629 (PMC9571735; doi:10.3390/jcm11195629)
Supplement: Supplementary file 1 [file jcm-11-05629-s001.zip › jcm-1888253-supplementary.pdf]

1 SUPPLEMENTARY INFORMATION S1: ASSESSMENT OF PREOPERATIVE COGNITIVE IMPAIRMENT

Before surgery, all patients performed a neuropsychological test battery consisting of paper-based and computerized subtests (CANTAB, Cambridge Cognition Ltd., UK). Tests include mean correct latency from the Simple Reaction Time (SRT, processing speed), number of correctly remembered items in the free recall task (VRM free recall) as well as the number of correctly recognised items after delay on the Verbal Recognition Memory test (VRM recognition, verbal memory), span length in the Spatial Span task (SSP, working memory), the first trial memory score from the Paired Associate Learning test (PAL, visual memory), completion time of the Trail-Making-Test-B (TMT-B, executive functions) and completion time for the Grooved Pegboard test (GPT, fine motor skills).

For these test parameters, we calculated Z scores of the baseline measurement in each test parameter in relation to the baseline measurements compared to an age-matched non-surgical control group and applied the Rasmussen criteria for postoperative cognitive dysfunction (two Z scores or a compound Z score < -1.96) to define preoperative cognitive impairment [1].

2 SUPPLEMENTARY INFORMATION S2: ASSESSMENT OF FRAILTY STATUS

The Frailty Phenotype is based on the Fried Criteria [2]. Five criteria were considered: weight loss, exhaustion, low physical activity, muscle weakness and slow gait speed. A patient was classified as frail if at least three criteria were met and as pre-frail if one or two criteria were met.

Weight loss was defined as either > 3 kilograms (kg) within the last 3 months (according to Mini Nutritional Assessment (MNA) Question B [3]) OR ≥ 5% of body weight within the last year (according to Study of Osteoporotic Fractures (SOF) index [4]).

Exhaustion was either indicated through the SOF index OR question 13 of the GDS (both surrogates).

Low Physical Activity was defined as < 15 points on item 6 of the Barthel Index [5-7].

Muscle Weakness was defined through handgrip strength (HGS). The cutoffs are based on sex and body mass index [2], scoring the average of three trials from the dominant hand (if information was available, otherwise the best mean value left or right was used).

Male:	Female:
BMI ≤24: ≤29 kg	BMI ≤23: ≤17 kg
BMI 24.1-26: ≤30kg	BMI 23.1-26: ≤17.3kg
BMI 26.1-28: ≤30 kg	BMI 26.1-29: ≤18 kg
BMI >28: ≤32kg	BMI >29: ≤21kg

BMI: Body-Mass-Index

Impairment of Gait Speed was defined as slowness in Timed-Up and Go (TUG) test (cut-off ≥ 10 seconds [8]).

3 SUPPLEMENTARY INFORMATION S3: FURTHER INFORMATION ON THE MODELS APPLIED

3.1 Fast-and-frugal Trees (FFTrees)

Fast-and-frugal trees are minimal binary classification trees that are constrained in terms of their structure: Each node in the tree is either an exit node (resulting in a decision or classification) or it has two child nodes. There is one exit node on each level below the root node with the exception of the lowest level that has two exit nodes. To classify cases with this tree, exactly one cue value is looked up on every level of the tree and the comparison of this value with a threshold determines which path of the tree is followed. As there are exit nodes on every level, each step can either result in an immediate decision or in the need to look up the subsequent cue value on the level below. A fast-and-frugal tree is thus determined by the order of cues whose values are looked up, the thresholds used, and the type of exit node (positive or negative classification) on each level.

Various algorithms have been proposed for the construction of fast-and-frugal trees [9-12]. Here, we chose two algorithms that proved most competitive in achieving a high balanced accuracy in [11], the ifan algorithm (FFTi) and the dfan algorithm (FFTd). Both algorithms used balanced accuracy as goal criterion and determined cue thresholds and directions so that decisions based on a single cue alone would achieve the highest possible balanced accuracy in the training sample. The FFTi algorithm sorts cues based on this measure, compares all possible exit structures with this determined cue order and chooses the one achieving the highest balanced accuracy in the training set (see [11] for details). The FFTd algorithm, in contrast, takes previous levels of the tree into account when determining the best cue for subsequent levels and re-calculates the performance of cues taking the cases into account that are already classified in previous levels. This results in a much more extensive search and a longer calculation time. In [11], had a similar predictive performance with differences between datasets in the competition.

3.2 Unconstrained Classification Trees (UDT)

A natural point of comparison for highly constrained fast-and-frugal trees are unconstrained classification trees (UDT) based on CART. CART [13] is one of the most popular algorithms for the construction of binary classification trees. The algorithm starts with a root node and chooses a cue and threshold to split the data into two subsets, resulting in two child nodes. The criterion for determining cue and threshold is the highest possible reduction in impurity as measured by the gini index. Impurity is minimal when each of the resulting nodes contains cases of one type alone and is at the highest point when both classes are evenly represented. The algorithm is then applied recursively to all resulting nodes until a stopping criterion is reached for all remaining nodes that become exit nodes. The stopping criterion is a combination of requirements for further splits: The process stops if there are less than 20 cases left in a node or the split does not result in a minimum improvement (with the complexity parameter set to 0.00001). To aim for a good performance in terms of balanced accuracy, we weighted misclassifications of positive cases higher than the misclassification of negative cases (based on the ratio of negative to positive cases in the training set). We used the rpart package in R [14] that implements most of the algorithms contained in [13].

3.3 Logistic Regression (LogReg)

The name-giving logistic function transforms unbounded continuous values into the interval between 0 and 1. The weighted sum of cue values is thus non-linearly transformed into a prediction of the probability of a case belonging to the positive class. In contrast to the tree-building algorithms, the resulting model will always require all cue values for predicting a novel case, unless cue weights are

predicted to be exactly zero. In addition to cue weights, the model requires a threshold to transform probability estimates into predictions.

Supplementary Table S1. : Overview over surgical sites of the surgical procedures (n=394)

**Surgical Site**	**Frequency (Percentage)**
Eyes	31 (7.9%)
Nose and the Paranasal Sinuses	8 (2.0%)
Face and in the Oral Cavity	13 (3.3%)
Pharynx Larynx and Trachea	3 (0.8%)
Lung and Bronchus	6 (1.5%)
Blood Vessels	12 (3.0%)
Lymphatic Vessels	9 (2.3%)
Digestive Tract	124 (31.5%)
Urinary Organs	9 (2.3%)
Female Genital Organs	35 (8.9%)
Jaw and Craniofacial Bone	8 (2.0%)
Locomotor System Organs	63 (16.0%)
Mamma	3 (0.8.%)
Skin and Underskin	12 (3.0%)
Nervous System	33 (8.4%)
Endocrine Glands	8 (2.0%)
Diagnostic Procedures (in general anaesthesia)	16 (4.1%)
Ears	1 (0.3%)

4 REFERENCES

1. Rasmussen, L. S. *et al.* The assessment of postoperative cognitive function. *Acta Anaesthesiol Scand* **45**, 275-289 (2001). https://doi.org:10.1034/j.1399-6576.2001.045003275.x
2. Fried, L. P. *et al.* Frailty in older adults: evidence for a phenotype. *The journals of gerontology. Series A, Biological sciences and medical sciences* **56**, M146-156 (2001). https://doi.org:10.1093/gerona/56.3.m146
3. Vellas, B. *et al.* The Mini Nutritional Assessment (MNA) and its use in grading the nutritional state of elderly patients. *Nutrition (Burbank, Los Angeles County, Calif.)* **15**, 116-122 (1999). https://doi.org:10.1016/s0899-9007(98)00171-3
4. Ensrud, K. E. *et al.* A comparison of frailty indexes for the prediction of falls, disability, fractures, and mortality in older men. *Journal of the American Geriatrics Society* **57**, 492-498 (2009). https://doi.org:10.1111/j.1532-5415.2009.02137.x
5. Mahoney, F. I. & Barthel, D. W. FUNCTIONAL EVALUATION: THE BARTHEL INDEX. *Md State Med J* **14**, 61-65 (1965).
6. Rockwood, K., Andrew, M. & Mitnitski, A. A comparison of two approaches to measuring frailty in elderly people. *The journals of gerontology. Series A, Biological sciences and medical sciences* **62**, 738-743 (2007). https://doi.org:10.1093/gerona/62.7.738
7. Siscovick, D. S. *et al.* Exercise intensity and subclinical cardiovascular disease in the elderly. The Cardiovascular Health Study. *American journal of epidemiology* **145**, 977-986 (1997). https://doi.org:10.1093/oxfordjournals.aje.a009066
8. Podsiadlo, D. & Richardson, S. The timed "Up & Go": a test of basic functional mobility for frail elderly persons. *Journal of the American Geriatrics Society* **39**, 142-148 (1991). https://doi.org:10.1111/j.1532-5415.1991.tb01616.x
9. Martignon, L., Katsikopoulos, K. V. & Woike, J. K. Categorization with limited resources: A family of simple heuristics. *Journal of Mathematical Psychology* **52**, 352-361 (2008).
10. Luan, S., Schooler, L. J. & Gigerenzer, G. A signal-detection analysis of fast-and-frugal trees. *Psychol Rev* **118**, 316-338 (2011). https://doi.org:10.1037/a0022684
11. Phillips, N., Neth, H., Woike, J. K. & Gaissmaier, W. FFTrees : A toolbox to create, visualize, and evaluate fast-and-frugal decision trees. *Judgment and Decision Making* **12** (2017).
12. Woike, J. K., Hoffrage, U. & Martignon, L. Integrating and testing natural frequencies, naïve Bayes, and fast-and-frugal trees. *Decision* **4**, 234-260 (2017). https://doi.org:10.1037/dec0000086
13. Breiman, L., Friedman, J.H., Olshen, R.A., & Stone, C.J. . *Classification And Regression Trees*. 1st edn, (Routledge, 1984).
14. Recursive Partitioning and Regression Trees v. R package version 4.1-16 (https://CRAN.R-project.org/package=rpart, 2019).
